# A Commentary on Realities of Developing COVID-19 Vaccines Discussed through the Global Health Safety Perspective

**DOI:** 10.3390/vaccines9030274

**Published:** 2021-03-18

**Authors:** Wedad Saeed Al-Qahtani, Fatmah Ahmed Alsafhi

**Affiliations:** 1 Department of Forensic Sciences, College of Criminal Justice, Naif Arab University for Security Sciences, Riyadh 11452, Saudi Arabia; 2 Department of Biology, College of Science, Princess Nourah Bint Abdulrahman University, Riyadh 11452, Saudi Arabia; faalsafhi@pnu.edu.sa

**Keywords:** COVID-19, SARS-CoV-2, vaccine, realities, possible concerns, health safety perspective

## Abstract

SARS-CoV-2 (or simply COVID-19) remains to be a global pandemic issue affecting millions, thus urging the world’s scientific community to develop efficient vaccine and design adequate measures of disease control. Currently, the most economically viable solution to infections and viruses is vaccination, despite the possible concerns about side effects from implementing quickly developed vaccine. The current commentary intends to explain the health and safety related to COVID-19 vaccines via a prism of global health safety. Scientists across the globe, along with companies from both public and private sectors, have predictably arranged cooperative programs to learn about COVID-19, along with taking simultaneous steps on devising vaccine and preparing effective treatments plans. Presently, several clinical trials to approve the efficiency of proposed vaccine solutions have been made successfully. Global health safety concerns on vaccine’s efficiency such as high costs of production, provision of vaccine to developing countries, and its influence on the global economy are addressed. This commentary reflects on current efforts related to the development of vaccine against COVID-19, which currently affects the global health status and economy. In addition, the commentary aims at addressing potential risks related to the development of COVID-19 vaccine from the global health safety perspective.

## 1. Introduction

The new coronavirus, also widely known as COVID-19, presumably came from Wuhan, China, starting in 2019. Wild bats were identified as potential carriers of the virus. Across generations of species, the virus eventually infected humans, spreading from individual to individual as an airborne virus. Historically, over the two last decades, there have been three major waves of coronavirus outbreaks initially transmitted from lower-order vertebrate: SARS CoV lasted between 2002 and 2003; MERS-CoV started and ended in 2012, and SARS-CoV-2, emerged in 2019 and is still prevalent in the present day. The preservation of various coronaviruses in wild bats (such as SARS-related CoV, i.e., Coronaviruses causing Severe Acute Respiratory Syndrome (SARS)) as well as occasional transmissions across species that eventually affect humans beings indicates that similar cases of global outbreaks may occur in the future [1].

After the initial outbreak in November 2019, COVID-19 has managed to penetrate 188 countries and 25 jurisdictions around the world. The governments and the WHO have been taking measures on contain the disease spread. However, the highly infectious essence of COVID-19 makes it particularly complicated. As per11:15 am CET 118,058,503 COVID-19 cases have been confirmed globally. In addition, 2,621,046 deaths have been recorded by the World Health (WHO). Moreover, 300,002,228 doses of vaccine had been administered as of 9 March 2021 [2].

Undoubtedly, the world community has an exclusive need in access safe and valid vaccines to improve the immunity of millions of potential victims of COVID-19 and reduce the global rates of morbidity and mortality caused by SARS-CoV-2. The broad geography of impact along with the global society’s need for efficient vaccine would definitely require multi-step strategy on developing a truly valid solution. Cooperative alliances between biotechnological researchers and pharmaceutical agencies specialized in vaccine production will be critically important during the pandemic [3]. The comprehensive roadmap of developing a vaccine against COVID-19 will demand close collaboration between industrial sectors, public administration, and scientists with each domain contributing to the whole situation. The commentary will reflect on a recently established collaborative program, namely the ACTIV (Accelerating COVID-19 Therapeutic Interventions and Vaccines) that represents a partnership between public and private interest groups which demonstrate the global solidarity in fighting the pandemic in a tough historical time.

The commentary will reflect on the topic of the global health safety of the current COVID-19 vaccines by addressing three critical elements: (1) risks associated with vaccine studies; (2) vaccine production and its efficiency; and (3) using neutralizing antibodies (nAbs) produced from individuals who have recovered from coronavirus and perspectives of this practice.

## 2. COVID-19 Vaccines and Global Health Safety Concerns

The recent emergence and spread of COVID-19 have underlined the necessity of developing-vaccines to fight emerging infectious diseases (EIDs). The problem is that vaccine producers have been negatively affected by the high cost of research and development (R&D) projects, research-related risks, and EID-vaccines have gained a lot of recognition in pandemic preparedness. In turn, officials and nonprofit organizations found themselves restricted in ensuring timely responses to these problems, eventually influencing overall production and regular logistics of vaccines [4]. Therefore, vaccine development becomes problematic even in the face of the global threat.

From the current limited financial perspectives, the expected economic returns from developing EID vaccine seems to be negative in relation to the cost invested. This means that the private sector alone will be unable to meet the global need for efficient vaccines without assistance from the public domain. The financial investment into an EID vaccine and realization of possible solutions are affected by increased price. Therefore, improved cooperation between public and private sectors, along with subscription options for receiving annual individual donations for accessing the list of available vaccines will be decisive [4].

Despite the assumption that current technologies would secure quick solutions against global infections, development costs are still of critical significance [5], especially for pharmaceutical projects. According to estimations by Pronker et al. [6], the development of a new vaccine would cost approximately between $200–and 900$ million. Vaccine approval, which usually takes place in 6-11% of all clinical trials, can also be associated with essential risks [4,5,7]. Regulatory issues are specifically noticeable in the creation of EID vaccines, as proper vaccine options are often unavailable during outbreaks, which makes the achievement of vaccine’s health safety and overall efficiency complicated. Hence, the development of EID-related vaccines is currently a technologically conservative strategy of a more reactive nature [8].

## 3. Risk Related to COVID-19 Vaccine Studies

Even though COVID-19 causes mainly mild symptoms in many infected people, there are individuals who seriously suffer from the disease. In selected cases, the virus can lead to lethal outcomes. The most vulnerable populations remain older people and people with preexisting and chronic medical complications, including cardiovascular problems, pulmonary complications, diabetes, and others [6,7].

Defining at-risk populations and profiles of victims is a crucial work that predetermines administrative decision-making and predicts the efficiency and outcomes of potential COVID-19 vaccine programs [4,6]. The safety concern arises when the vaccine has not been confirmed previously by regulatory bodies, while the amount of available vaccination options against COVID-19 comes from suspicious unconfirmed technologies and/or agencies. As noted by Byram Bridle, a credited viral immunologist from the University of Guelph, Canada, the creation of a COVID-19 vaccine during almost a calendar year, i.e., relatively quickly, has been an unheard case. This sounds disturbing since Bridle’s project was sponsored to design a new efficient vaccine. This trend has made an honorable and competent scientist in his area of specialization deeply concerned as the high pace of vaccine development could be accompanied by risks of compromised or insufficient evaluations of the vaccine designed. The unsafe and inefficient vaccine would be useless and, in some cases, even dangerous for use [8,9].

## 4. COVID-19 Vaccine Production and Its Efficiency

Scientific efforts have been mainly targeted on the production of vaccines to fight COVID-19 for the purpose of reducing the pandemic’s effects. To date, a vast number of produced vaccine options have applied the S-protein taken from SARS-CoV-2 [10,11,12]. By July 2020, the global pool of SARS-CoV-2 vaccines incorporates 158 potential vaccine options. Among registered options, 135 candidates remain to be preclinical or are still being explored. At the moment, vaccines titled as mRNA-1273 (Moderna, Cambridge, MA, USA), Ad5-nCoV (CanSino Biologicals, Tianjin, China), INO-4800 (Inovio, Inc., Plymouth Meeting, PA, USA), LV-SMENP-DC, Pathogen-specific aAPC (ShinzenGeno-Immune Medical Institute, Shenzhen, China), and ChAdOx1 (University of Oxford, Oxford, UK) have been put at the first and second stages of clinical trials [3,13].

All development programs aim at producing a vaccine that can enable the synthesis of S-protein neutralizing antibodies in the bodies of vaccinated COVID-19 individuals. Research has identified that there is either a restricted or zero cross-neutralization when comparing the serum of both SARS-CoV and SARS-CoV-2. This implies that successful recovery from one disease is not a guarantee that a subject will be safe from other infectious disease [3]. In addition, the vaccines placed in the special conduit have been developed on the ground of inactive or weakened-to-live-conditions viruses, protein sub-units, virus-like particles (VLP), viral vector (replicating and non-replicating), DNA, RNA, nanoparticles, and other simulated elements. Each component represents exclusive advantages, but also poses unpredictable challenges [10,11,12,13,14,15]. To minimize uncertainty and make better use of human immune response, auxiliary technologies including AS03 (GSK, Brentford, UK), MF-59 (Novartis, Basel, Switzerland), CpG 1018 (Dynavax, Emeryville, CA, USA) and others can be applied by scientists to develop the necessary vaccines [16]. In addition, the method based on immuno-informatics is applied for identifying epitope in terms of manufacturing SARS-CoV-2 vaccine options. The technology is utilized to define the considerable cytotoxic T-cell and B-cell epitopes in the context of studying viral proteins [3,7,10,14,15,16,17].

Currently, there are several COVID-19 candidate vaccines in Stage III trials. These vaccines are based on an mRNA spike protein carried via lipidic microparticles. In addition, two of the vaccines have gone beyond Stage III trials and have been authorized by WHO. On 31 December 2020, WHO approved the Comirnaty mRNAbased vaccine for emergencies, making the BioNTech/ Pfizer’s vaccine the introductory one to gain emergency authorization from WHO after the outbreak started in 2020 [18]. The mRNA’s vaccines from Moderna (known as mRNA-1273) and Pfizer (known as BNT162b2) indicated approximately 95% efficiency in the prevention of symptomatic COVID-19 following the second vaccination with the final outcomes of the Stage III trial registering 45539 participants that showed approximately 95% efficiency for Pfizer while 30,000 participants showed approximately 94% effectiveness for Moderna. The mRNA’s technology is a mechanism of offering the body genetic instructions to synthesize the COVID-19′s spike protein. This idea entails priming the immune system to form a defensive immune response in case one encounters the SARS-CoV-2. Additionally, the Food and Drug Administration (FDA) has authorized the vaccines from Moderna and Pfizer. Experts have validated the three vaccines as effective and safe.

In accordance with the last WHO’s updated data, the mRNA vaccine from Pfizer-BioNTech is more effective and safer. Nonetheless, there are particular people for whom vaccines are not recommended as a result of contradictions or limited data. Presently, these individuals include the majority of pregnant women, people having severe allergies’ history, and international travellers not included in a prioritized group as well as children below 16 years. Health workers have been prioritized in vaccination efforts due to their high exposure risk, followed by the older adults, and then immunize the larger population [2].

Other potential vaccines presently in Stage III trials are hinged on spike protein’s DNA carried through adenoviruses. Such vaccines include Russia’s Gamaley Res. Inst. Sputnik and China’s Can Sino Ad5-nCoV vaccines that have obtained limited approval and have vaccinated partial populations. Moreover, in spite of the preliminary outcomes as well as well-recorded immunogenicity, the Stage III trial of the University of Oxford/ AstraZeneca ChAdOx1 vaccine offered provocative although contradictory outcomes that need further studies.

On 15 February 2021, the World Health Organization authorized two Oxford/ AstraZeneca’s COVID-19 vaccine’s versions known as ChAdOx1-S for emergencies, allowing their roll out worldwide through COVAX [2]. These vaccines are manufactured by the Indian Serum Institute and the Republic of Korea’s AstraZeneca-SKBio. The vaccine from Oxford has a genetic sequence as the surface spike’s protein. After the entrance of the vaccine into human cells, it utilizes the genetic code for the production of the coronaviru’s surface spike proteins. This triggers immunity, preparing the immunity system to handle COVID-19 if it infects the body later [2]. Moreover, the vaccine was approved for emergencies in the United Kingdom in December 2020. It has also received approval from over 40 countries globally. ChAdOx1-S has shown 82.4% effectiveness after the second vaccination and is suited for middle- and low-income states because of its affordable storage needs [2].

On 27 February 2021, the Johnson & Johnson vaccine was also approved for emergency usage, which made it the third COVID-19 vaccine from the United States (US). Additionally, it was the first to show effectiveness and safety after receiving a single dose as opposed to two doses for the other vaccines. Furthermore, clinical trials have indicated a 72 % efficacy rate for one dose [2].

Generally, even though these COVID-19 vaccines are promising in the prevention of deaths and severity of infections, they cannot offer 100% protection. In addition, the whole immunity process normally takes approximately 2 weeks following a vaccine’s second dosage. That is the reason why individuals can still get infected with the new COVID-19 and become sick in case of exposure shortly after receiving the vaccines [2].

## 5. Neutralizing Antibodies (nAbs) Produced from Individuals Recovered from Coronavirus and Perspectives of This Practice

Some research projects focusing on SARS-CoV and MERS-CoV have determined that many components (for example, S1-NTD, RBD, S2) located in S-proteins might be exploited as a basis for generating nAbs. Nevertheless, RBD-directed antibodies have a better chance to eliminate infection when divergent virus strains are used. It is presumed that the RBD of SARS-CoV-2 can be used as a key target for manufacturing strong neutralizing antibodies [18,19,20,21,22,23,24]. Mixtures incorporating antibodies centered on RBD and other areas in the selected S-protein might enhance the scope of action and strength of nAbs intensively in neutralizing SARS-CoV-2 and its mutating strains. Indeed, human serum derived from recovering patients has been applied to manage COVID-19 symptoms, yet outcomes of such an approach are equivocal. SARS analysis revealed that there could be non-nAbs that hit RBD-unrelated areas in the S-protein, which might provoke the so-called antibody-dependent enhancement (ADE). Among negative effects, viral infectiousness, disease progress, and other undesired immune reactions are mentioned [18,19]. On the other hand, some nAbs have demonstrated positive results on cross-reactivity or cross-neutralization of the progression of SARS-CoV-2, even though in vitro [23,24]. In general, studies examining SARS-CoV and/or MERS-CoV related to nAbs are recommended to include detailed guidelines for the quick manufacturing of nAbs targeting SARS-CoV-2.

## 6. Types of Candidate Vaccines and the Side Effects Observed for SARS-CoV-2 Vaccine

The vaccine against SARS-CoV-2 should achieve the following outcomes: to reduce adverse immunopotentiation, be appropriate for use by adults above 60 or with primary features of hypertension or diabetes, be appropriate for durable accumulation, and be suitable for adult healthcare professionals [25]. Fifty-two vaccines against SARS-CoV-2 have been reported to be undergoing clinical trials as of 4 November 2020. Licensures have been provided to four types of vaccines: Viral Vector-Based, Live-Attenuated, protein subunit, and Inactivated (i.e., the killed whole-cell antigens of the virus). Moreover, two types of this vaccine-RNA & DNA-have not been given licensure [26].

Currently, the global focus is made on eliminating and preventing the COVID-19 pandemic. The permanent search for appropriate and reliable candidates for SARS-CoV-2 vaccines is impressive. The improvement in immunological reaction may be achieved by using auxiliary agents for protein vaccines. However, the large implementation of attenuated vaccines is basically related to the potential of pathogen reactivation and its subsequent toxicity [27]. Currently, safety, the lack of side effects, and the efficiency of all vaccine technologies are jeopardized.

Though some side effects of the vaccine resemble the COVID-19 symptoms, this disease cannot be triggered by the coronavirus vaccine. In addition, the vaccines are not infectious. The majority of individuals experience moderate or mild vaccine side effects within 1-2 days [28]. Nevertheless, some studies specified that if vaccine production were problematic, the generation of the lethal vaccine doses would be implemented by a single company or possess the analogous lot number. It should be noted that vendors and lot numbers of the vaccine differ. Therefore, it is strong evidence that this is not an issue in the production process [29]. Vaccine dissemination or warehousing on a small scale may lead to the occurrence of such issues as refrigerator errors. Nevertheless, in this case, insignificant side effects or fatal outcomes are reported in patients, who received vaccination in similar medical facilities. Another aspect is related to the fact that medical institutions, where the vaccinated patients died, did not inform about more adverse effects [28,29,30].

Furthermore, Guillain Barre syndrome and anaphylaxis are the common side effects of vaccines. Guillain Barre syndrome represents the most severe side effect caused by vaccination [26]. Nevertheless, symptom development is typically reported because muscle weakness occurs in the period of half a day to some weeks. The occurrence of anaphylaxis takes place after vaccination. The currently registered incidents demonstrate the long period in the intervals between patient vaccination and fatal outcome, excluding the potential for anaphylaxis. Current episodes prove to be sudden fatal outcomes, with the lack of reports of progressive cases. Thus, an adequate conclusion should inform that the causality between death and vaccination does not depend on the epidemiological findings exclusively. Moreover, the conclusion is reinforced by the lack of cases reported on these side effects in toddlers and infants, who get vaccinated alongside the elderly [30].

## 7. Conclusions

The widespread of COVID-19 changed global health priorities and realities of approaching vaccination routine. Development of vaccine in modern disturbing times is full of pitfalls. There are several critical health safety concerns regarding production and use of coronavirus vaccines. For instance, the rapid development of the vaccine might compromise its assessment before use, increasing risks of unexpected adverse effects since the vaccine itself does not give full protection from infection. In addition, health and safety is the primary field of business of the healthcare companies involved in healthcare which have to confront the economic challenges, and their interests can be beyond securing health, unfortunately. Nevertheless, it is evident that private-public partnerships along with the efforts of the scientific community, make it possible to create efficient COVID-19 vaccines that could be useful and safe in the long-term perspective.
